# Primary hepatic non-Hodgkin’s lymphoma with rectal cancer: A case report

**DOI:** 10.3892/ol.2014.2673

**Published:** 2014-11-05

**Authors:** GUO-BIN WU, CHAO-YUAN HUANG, SHAN HUANG, HAI-MING RU, BANG-DE XIANG, WEI-PING YUAN, FEI-XIANG WU, JIAN-YONG LIU, ZHI-MING ZHANG, LIANG MA, ZU-SHUN CHEN, YIN-NONG ZHAO, LE-QUN LI

**Affiliations:** Department of Hepatobiliary Surgery, Affiliated Tumor Hospital and Oncology School of Guangxi Medical University, Nanning, Guangxi 530021, P.R. China

**Keywords:** non-Hodgkin’s lymphoma, liver, primary liver lymphoma, rectal cancer

## Abstract

Primary hepatic non-Hodgkin’s lymphoma (NHL) is an extremely rare disease that is commonly neglected as a possible diagnosis. The present study reports the case of a middle-aged male with chronic hepatitis B in which primary hepatic NHL and rectal cancer occurred simultaneously. A large solitary tumor in the left lobe of the liver was incidentally detected on routine examination prior to the laparoscopic resection of the rectal cancer. Laparoscopic resection of the rectal cancer and a liver biopsy were performed simultaneously. The pathology revealed that the hepatic tumor was NHL and that the rectal cancer was adenocarcinoma. Systemic staging revealed no evidence of nodal or bone marrow involvement, therefore, primary hepatic lymphoma (PHL) was diagnosed. PHL associated with rectal adenocarcinoma is extremely rare and to the best of our knowledge, has never been reported. At present, the cause and most effective therapy for the condition remain unclear.

## Introduction

Primary hepatic lymphoma (PHL) is an extremely rare malignancy. PHL is defined as an extranodal lymphoma of the liver without involvement of any other organs. PHL constitutes 0.4% of cases of extranodal non-Hodgkin’s lymphoma (NHL), and comprises ~0.01% of all NHL (1). PHL occurs in males twice as often as in females, and the usual age at presentation is >50 years (2). The symptoms are usually non-specific, however, the most common symptom is abdominal pain. Additionally, laboratory tests and cancer markers are non-specific. Liver biopsies remain the most valuable tool for the diagnosis of PHL (3). The CHOP (cyclophosphamide, doxorubicin, vincristine, prednisone) chemotherapy regimen is the standard treatment and the prognosis of patients with PHL is associated with PHL subtypes (4). The current study presents an unusual case of primary NHL with rectal cancer. A review of the literature with regard to the clinical features, diagnosis and management of PHL is also provided. Written informed consent was obtained from the patient for publication of this case study.

## Case report

In November 2012, a 56-year-old male presented to the Affiliated Tumor Hospital and Oncology School of Guangxi Medical University (Nanning, Guangxi, China) with a 3-month history of bloody stools. The patient exhibited no symptoms of a fever, night sweats, nausea, vomiting, chest pain, abdominal pain, diarrhea, loss of appetite, changes in bowel habits or weight loss. A physical examination showed no notable results. No superficial lymphadenopathy was present.

The laboratory results included a hemoglobin level of 146.00 g/l and a white cell count of 4.79×10^9^/l, with a normal differential. The levels of alanine aminotransferase, aspartate aminotransferase, alkaline phosphatase and lactate dehydrogenase were also within normal limits. The serum tumor marker results included a cancer antigen 125 level of 22.49 U/ml, and a cancer antigen 15-3 level of 8.92 U/ml, as well as normal levels of serum α-fetoprotein and carcinoembryonic antigen. Serology was positive for the hepatitis B virus (HBV), and negative for the hepatitis C virus (HCV) and the human immunodeficiency virus.

**Figure 1:**
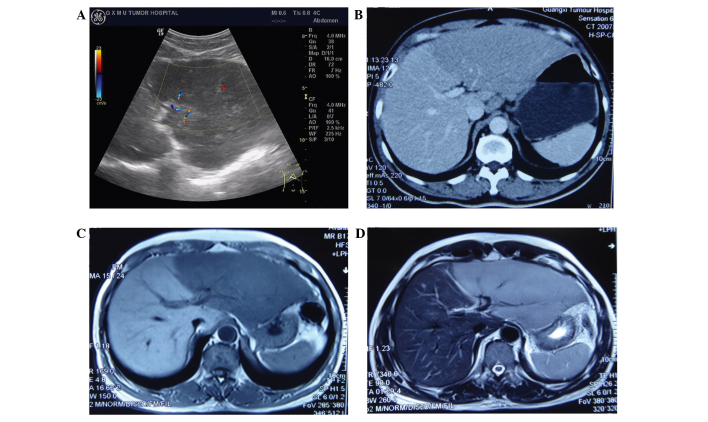
Imaging studies of the left lobe of the liver by (A) ultrasound examination, (B) computed tomography, and (C) T1-weighted imaging (WI) and (D) T2WI by magnetic resonance imaging.

Imaging studies of the left lobe of the liver showed abnormal increases with smooth edges, and mixed iso- and hypoechogenicity in the ultrasound examination. Hypodensity in the pre-contrast phase and no enhancement in the post-contrast phase was observed on computed tomography (CT). Hypointensity was observed on T1-weighted imaging (WI) and hyper-intensity on T2WI by magnetic resonance imaging (MRI) (Fig. 1). Radiography and CT did not reveal any mediastinal and abdominal lymphadenopathy. The pancreas, spleen, and biliary tract were normal.

**Figure 2:**
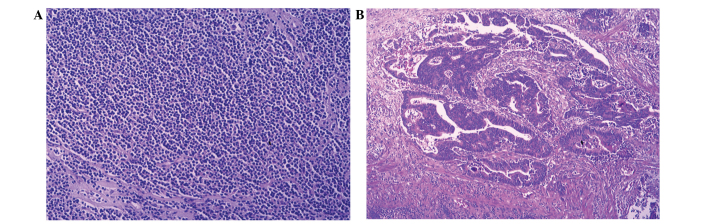
(A) Pathological result of the biopsy from the lesion of the left lobe of the liver. (B) Pathological result of the resected rectal cancer.

Colonoscopy showed a tumor in the bowel wall growing out from the anus by 3–5 cm, with surface erosion, and pathological analysis of a biopsy specimen that was obtained revealed a moderately-differentiated adenocarcinoma. Histological analysis following ultrasound-guided biopsy of the lesion in the left lobe of the liver showed diffuse infiltrates and small to intermediate atypical cells consistent with lymphoma (Fig. 2), Immunostaining of the tumor cells showed reactivity for cluster of differentiation 79a, pax-5, light chain λ and B-cell lymphoma-2.

Bone marrow biopsy showed normal cellularity with maturing trilineage hematopoiesis in normal proportions. No histological or immunophenotypical evidence of B-cell lymphoma was present.

The patient was diagnosed with rectal adenocarcinoma with primary liver lymphoma, tending towards a large B-cell lymphoma of the mucosa-associated lymphoid tissue-type, given that no additional foci of lymphoma were found anywhere else in the body. Laparoscopic resection of the rectal cancer and liver biopsy specimens was performed simultaneously. The pathology of the hepatic tumor confirmed the previous diagnosis. The patient received six cycles of cyclophosphamide-doxorubicin-vincristine-prednisone (CHOP) chemotherapy (750 mg/m^2^ cyclophosphamide on day 1; 50 mg/m^2^ doxorubicin on day 1; 1.4 mg/m^2^ vincristine on day 1; and 100 mg/m^2^ prednisone on days 1–5) for six months, subsequent to the surgery. CT showed no metastasis of the rectal cancer and no change to the PHL at six months post-chemotherapy. To date, the patient’s condition remains stable.

## Discussion

Although secondary liver involvement of lymphoma in the advanced stage is common, PHL, which is defined as lymphoma either confined to the liver or having major liver involvement, is extremely rare (1,5) and represents <1% of all extranodal lymphomas (6).

The exact cause of PHL remains unclear. A number of recent studies have shown a higher prevalence of HCV or HBV infection in PHL patients (4,7,8). Hepatitis C is found in 40–60% of patients with PHL (2). Other studies have hypothesized that PH may be associated with cirrhosis and immunosuppressive drugs (9,10). However, the patient in the present study was positive for HBV and also diagnosed with rectal cancer, but the association between the PHL and rectal cancer was not clear. No cases exhibiting this combination have been reported in the past.

PHL occurs in males twice as often as in females, and the usual age at presentation is 50 years (2). The symptoms are usually non-specific, however, the most common symptom is abdominal pain. Hepatomegaly also occurs frequently, while jaundice is an occasional finding upon physical examination (1,11). Additionally, laboratory tests and cancer markers are non-specific.

Upon the imaging of PHL, certain characteristics are shown. Upon ultrasound examination, the lesions are of mixed iso- and hypoechogenicity, with a hypoechoic rim. On CT scans, the lesions appear as hypodense in the pre-contrast phase and as rim enhancement in the post-contrast phase (12,13). On MRI, the lesions show hypointensity on T1WI and hyperintensity on T2WI. These imaging features differ from focal nodular hyperplasia, hepatocellular carcinoma, cholangiocarcinoma or metastases (14). The imaging examinations in the present study revealed the majority of the usual PHL features, such as hepatomegaly of the left lobe of the liver.

The final diagnosis of PHL relies on histological examination. Liver biopsies remain the most valuable tool for the diagnosis of PHL (3). The majority of cases of PHL are diffuse large B-cell lymphoma. Other histological subtypes of PHL include high-grade tumors (lymphoblastic and Burkett lymphoma; 17%), follicular lymphoma (4%), diffuse histiocytic lymphoma (5%), lymphoma of the mucosa-associated lymphoid tissue-type, anaplastic large-cell lymphoma, mantle cell lymphoma and T-cell-rich B-cell lymphoma (3). The present patient underwent a liver biopsy twice and was diagnosed with primary B-cell lymphoma, tending towards the B-cell lymphoma of the mucosa-associated lymphoid tissue-type, given that no additional foci of lymphoma were found anywhere else in the body.

The optimal therapy for PHL remains unclear and the outcomes are uncertain. Certain clinicians use surgery only, while others prefer chemotherapy alone or combined with radiotherapy (1). Although chemotherapy is used to treat the majority of patients, certain physicians undertake a multimodality approach, which also integrates surgery and radiotherapy (1). The CHOP regimen is the standard treatment for patients with diffuse large B-cell lymphoma. The addition of rituximab to the CHOP regimen, when given in eight cycles, augments the complete response rate and prolongs event-free and overall survival times in older patients with diffuse large B-cell lymphoma, without significantly increasing the clinical toxicity (15,16). The present patient received one cycle of CHOP chemotherapy following a laparoscopic resection of the rectal cancer. CT showed no metastasis of the rectal cancer and no change of the PHL at six months post-chemotherapy. At present, the patient’s condition is stable and is attending follow-up examinations to monitor any long-term effects.

In conclusion, PHL associated with rectal adenocarcinoma is extremely rare and to the best of our knowledge, has never reported. The cause of PHL remains unclear. Diagnosis of this condition is important, and if the clinical conditions are indicative of PHL, a liver biopsy should be obtained. As the optimal therapy is unclear, the overall survival rate for patients with PHL tends to be poor.
